# Disruption of maternal gut microbiota during gestation alters offspring microbiota and immunity

**DOI:** 10.1186/s40168-018-0511-7

**Published:** 2018-07-07

**Authors:** Donald D. Nyangahu, Katie S. Lennard, Bryan P. Brown, Matthew G. Darby, Jerome M. Wendoh, Enock Havyarimana, Peter Smith, James Butcher, Alain Stintzi, Nicola Mulder, William Horsnell, Heather B. Jaspan

**Affiliations:** 1 Institute of Infectious Diseases and Molecular Medicine, Department of Pathology, Division of Immunology, University of Cape Town, Cape Town, South Africa; 2 Institute of Infectious Diseases and Molecular Medicine, Department of Integrative Biomedical Sciences, Division of Computational Biology, University of Cape Town, Cape Town, South Africa; 3 Duke University, Durham, NC USA; 4 Ottawa Institute of Systems Biology, Department of Biochemistry, Microbiology and Immunology, Faculty of Medicine, University of Ottawa, Ontario, CA USA; 5 Institute of Microbiology and Infection, University of Birmingham, Birmingham, B15 2TT UK; 6 Laboratory of Molecular and Experimental Immunology and Neurogenetics, UMR 7355, CNRS-University of Orleans and Le Studium Institute for Advanced Studies, Rue Dupanloup, 45000 Orléans, France; 7 Department of Pediatrics and Global Health, University of Washington and Center for Global Infectious Disease Research, Seattle Children’s Research Institute, Seattle, WA USA; 8 Present Address: Center for Global Infectious Disease Research, Seattle Children’s Research Institute, Seattle, WA USA

## Abstract

**Background:**

Early life microbiota is an important determinant of immune and metabolic development and may have lasting consequences. The maternal gut microbiota during pregnancy or breastfeeding is important for defining infant gut microbiota. We hypothesized that maternal gut microbiota during pregnancy and breastfeeding is a critical determinant of infant immunity. To test this, pregnant BALB/c dams were fed vancomycin for 5 days prior to delivery (gestation; Mg), 14 days postpartum during nursing (Mn), or during gestation and nursing (Mgn), or no vancomycin (Mc). We analyzed adaptive immunity and gut microbiota in dams and pups at various times after delivery.

**Results:**

In addition to direct alterations to maternal gut microbial composition, pup gut microbiota displayed lower α-diversity and distinct community clusters according to timing of maternal vancomycin. Vancomycin was undetectable in maternal and offspring sera, therefore the observed changes in the microbiota of stomach contents (as a proxy for breastmilk) and pup gut signify an indirect mechanism through which maternal intestinal microbiota influences extra-intestinal and neonatal commensal colonization. These effects on microbiota influenced both maternal and offspring immunity. Maternal immunity was altered, as demonstrated by significantly higher levels of both total IgG and IgM in Mgn and Mn breastmilk when compared to Mc. In pups, lymphocyte numbers in the spleens of Pg and Pn were significantly increased compared to Pc. This increase in cellularity was in part attributable to elevated numbers of both CD4+ T cells and B cells, most notable Follicular B cells.

**Conclusion:**

Our results indicate that perturbations to maternal gut microbiota dictate neonatal adaptive immunity.

**Electronic supplementary material:**

The online version of this article (10.1186/s40168-018-0511-7) contains supplementary material, which is available to authorized users.

## Background

The gut microbiota during a critical window in infancy is key for immune development, establishment of oral tolerance, and mucosal barrier function [1]. The fetal gut has long been assumed to be sterile, with colonization occurring only at delivery [2]. Although controversial, this dogma has recently been challenged with the apparent identification of low abundance bacteria in fetal membranes, amniotic fluid, and placenta [3–8]. Regardless, maternal diet before and during pregnancy has been shown to influence offspring metabolism, as well as susceptibility to allergy and bacterial infections [9–11]. Postpartum, maternal factors continue to be important determinants of early infant colonization [2], including type of infant feeding [12]. Both breast milk bacterial composition [13] and human milk oligosaccharides (HMOs) are thought to influence infant gut microbiota. Breast milk has its own microbiota, the origins of which are not completely understood, but are believed to be partly due to translocation from the gut [14].

Antibiotics, although necessary in some cases, can lead to disruption of the commensal bacteria with lasting consequences [15, 16]. Maternal antibiotics during lactation have lasting metabolic and immunological consequences on offspring mediated presumably via alteration of the neonatal microbiome [17–20]. In germ-free mice, transient colonization of maternal intestines with *E. coli* during gestation causes intestinal innate immune alterations in the offspring, yet does not influence pup adaptive immunity [21]. Here, using oral vancomycin, which has low oral bioavailability, we show that alteration of maternal gut microbiome during gestation, nursing, or both has persistent effects on offspring gut microbiota and systemic adaptive immunity.

## Results

### Maternal gut microbiota during gestation and nursing differentially shape infant mouse intestinal microbiota

**Fig. 1:**
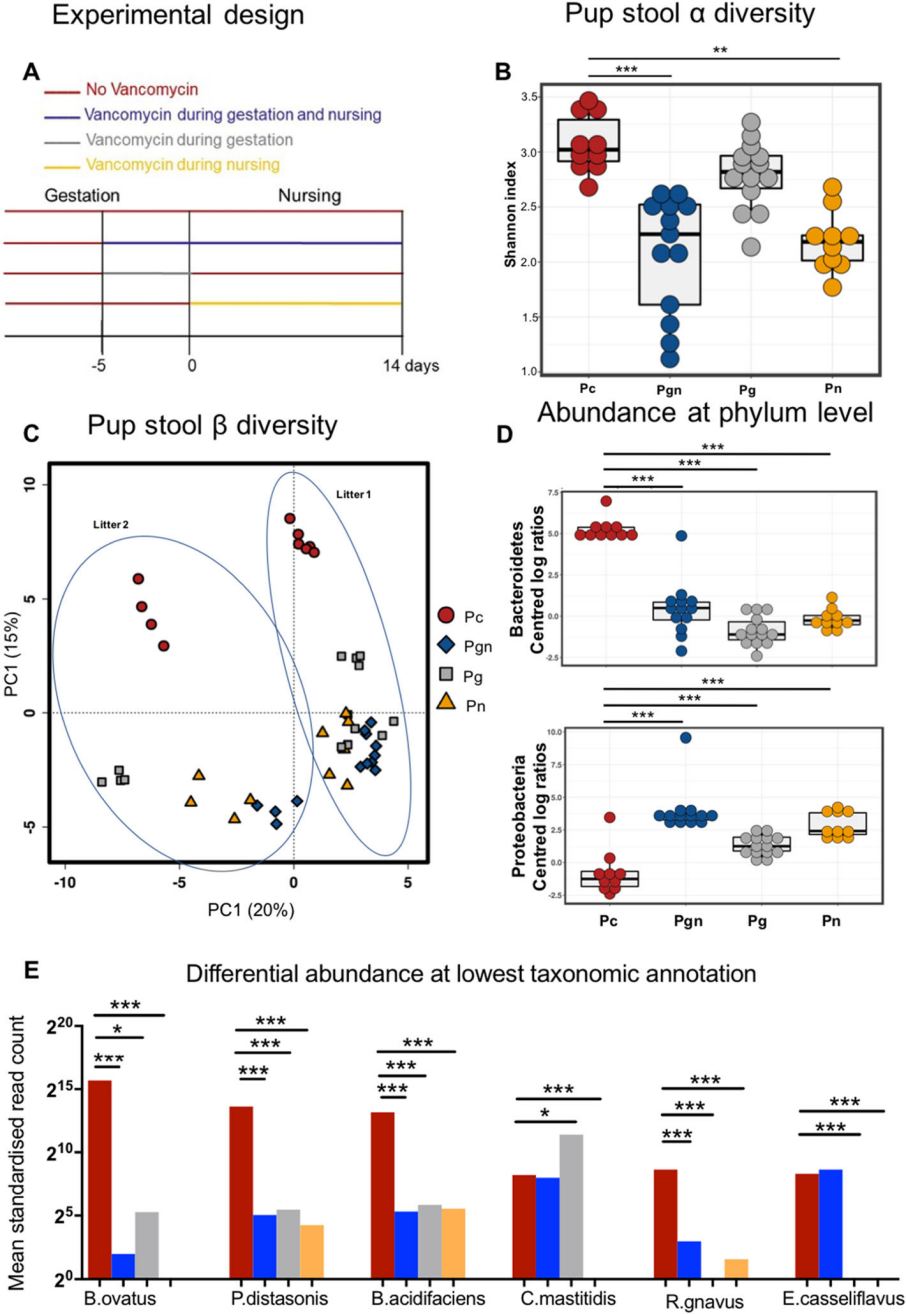
Maternal oral vancomycin profoundly alters pup intestinal microbiota composition. **a** Experimental setup: BALB/C mice received oral vancomycin (1 mg/mL) for 5 days prior to delivery (gestation: Mg), 14 days after delivery (nursing: Mn), or 5 days prior to delivery through 14 days of nursing (gestation plus nursing: Mgn). Control mice (Mc) were not exposed to vancomycin. Fecal samples were collected from both mothers and their offspring 14 days postpartum. **b** Shannon index of pup fecal microbiotas of pups born to control (Pc: brown), Mgn (Pgn: blue), Mg (Pg: gray), or Mn (Pn: yellow) dams. Boxplot shows 25th and 75th percentiles with a line at the median. **c** Principal coordinate analysis of pups’ fecal microbial β-diversity based on Bray-Curtis dissimilarity. **d** Significantly different taxa abundances at phylum level in pup fecal microbiota communities after centered log ratio data transformation (Kruskal-Wallis and Mann Whitney *U* test). **e** Significantly differentially abundant OTUs in pup fecal microbiota obtained from both MetagenomeSeq and Deseq2 analyses performed on data merged at the lowest taxonomic level. *B. ovatus*: Bacteroides ovatus, *P. distasonis*: Parabacteroides distasonis, *B. acidifaciens*: Bacteroides acidifaciens, *C. mastitidis*: Corynebacterium mastitidis, *R. gnavus*: Ruminococcus gnavus, *E. casseliflavus*: Enterococcus casseliflavus. Results were combined from two independent experiments. *n* = 6–10 pups per group per experiment. *p < 0.05, **p < 0.01, ***p < 0.001

To assess the impact of maternal gut microbiota during fetal development and nursing on infant microbiota, we treated pregnant dams with oral vancomycin for 5 days prior to delivery (gestation: Mg), for 14 days postpartum during nursing (nursing: Mn), or both (gestation and nursing: Mgn) and compared these to control dams (Mc) (Fig. 1a). We analyzed intestinal microbiota by sequencing the V6 region of the 16S rRNA gene of bacterial DNA from intracolonic fecal pellets obtained 14 days postpartum. Maternal oral vancomycin had distinct effects on maternal gut microbiota at 14 days postpartum, depending on the timing of treatment. Gut microbiota α-diversity was decreased in vancomycin-treated mothers compared to controls (Additional file 1: Figure S1A). Principal coordinate analysis (PCoA) of β-diversity (by Bray-Curtis dissimilarity) demonstrated that all antibiotic-treated dams clustered separately from Mc, although Mgn and Mn clustered together (Additional file 1: Figure S1B). Pup intestinal microbiota at 14 days postpartum also showed distinct changes associated with maternal vancomycin treatment. We observed significantly reduced α-diversity, in pups born to dams treated with vancomycin during nursing (Pn) or gestation plus nursing (Pgn) compared to pups born to control dams (Pc) (Fig. 1b). Further, PCoA analysis of β-diversity by Bray-Curtis dissimilarity demonstrated the existence of distinct microbial communities dependent on the timing of maternal exposure to vancomycin (Fig. 1c; Adonis *R*^2^ = 0.449, *p* = 0.001). Pg had microbiomes distinct from all other groups, suggesting that disruption of maternal gut microbiota with vancomycin during gestation only alters offspring microbiota differently as compared to vancomycin treatment during breastfeeding. In a PCoA including both maternal and pup microbiota, pup communities clustered closest to their own mothers’ communities (Additional file 1: Figure S1D).

As expected, at the Phylum level, stool of vancomycin-treated dams had higher relative abundance of Proteobacteria versus Mc (Additional file 1: Figure S1C). However, the relative abundance of the phylum Bacteroidetes (also gram negative) was decreased in all antibiotic-treated dams compared to Mc. This was also evident in pups from antibiotic-treated dams who displayed significantly reduced relative abundance of Bacteroidetes in their colonic contents as compared to control pups (adj *p* < 0.001 vs Pc for all intervention groups). In addition, Proteobacteria was significantly increased in pups born to vancomycin-treated dams regardless of timing compared to control pups (adj *p* < 0.001 vs Pc for all intervention groups Fig. 1d). Next, using both Deseq2 and metagenomeSeq, we identified several significantly differentially abundant taxa across the groups after merging at the lowest taxonomic annotation. *Bacteroides acidifaciens*, *Bacteroides ovatus*, *Ruminococcus gnavus*, and *Parabacteroides distasonis* were significantly less abundant in all intervention groups compared to Pc (adj *p* < 0.001 vs Pc for all except Pc vs Pg B ovatus *p* < 0.05), Fig. 1e). Additionally, *Corynebacterium mastitidis* was significantly increased in Pg compared to Pc but was undetectable in Pn. Moreover, *Enterococcus casseliflavus* was undetectable in Pg and Pn. Overall, these data suggest that maternal gut microbiota influence pup gut microbiota.

### Maternal gut microbiota impacts breastmilk and genital tract microbiota

**Fig. 2:**
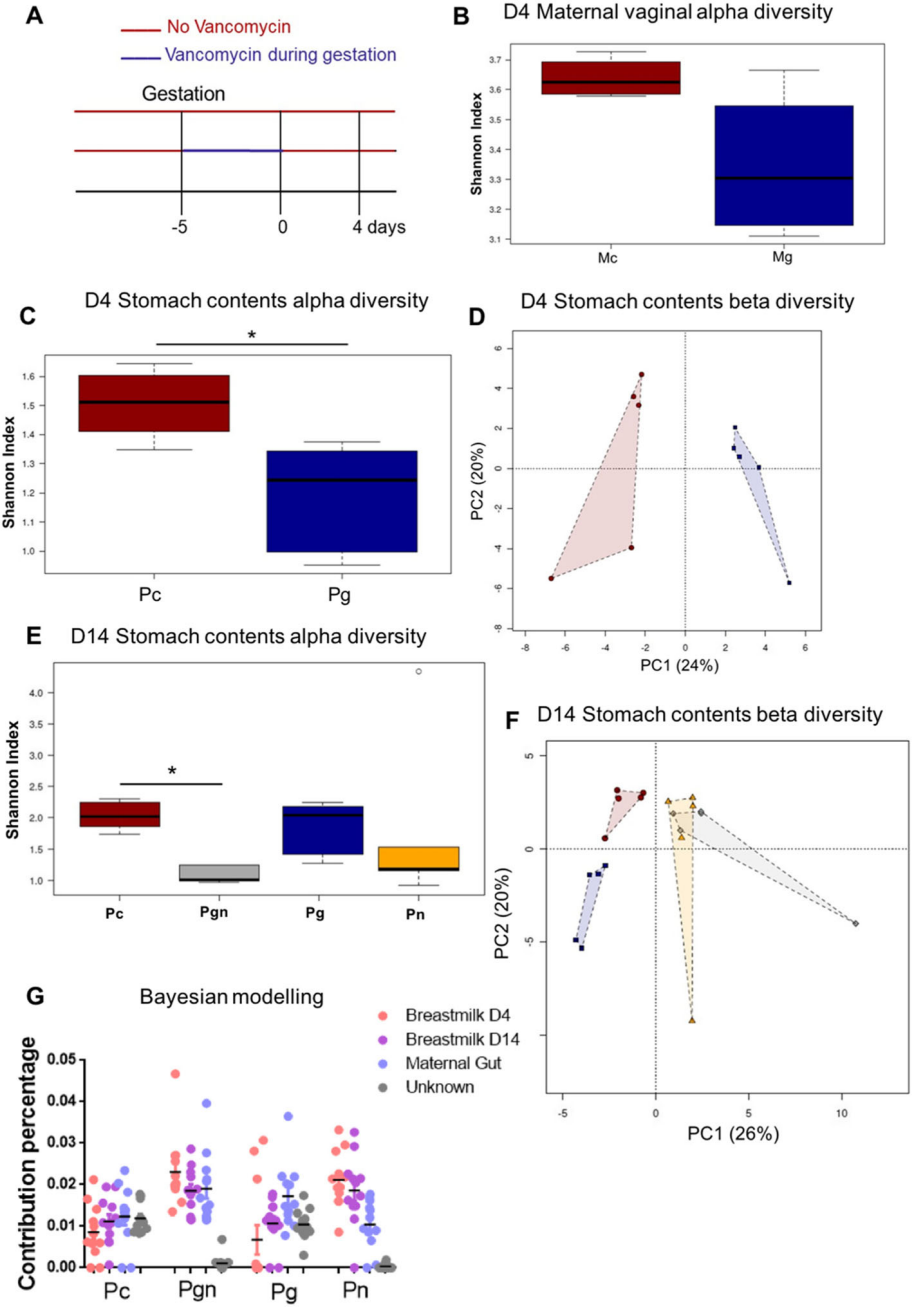
Maternal gut microbiota indirectly impacts genital tract and breastmilk microbiota. **a** Experimental setup: BALB/c mice were treated with vancomycin 5 days prior to delivery (Mg) or left untreated (Mc). Four days after delivery, mice were sacrificed and genital tract samples collected for 16S rRNA gene analysis. Stomach contents were also collected from pups’ stomach for microbiome analysis. **b** Shannon index of Mg (blue) versus Mc (red) genital tract microbiota. **c** Shannon index of stomach contents microbiota (collected from pups’ stomach 4 days after delivery). **d** Principal coordinate analysis of stomach contents microbiota day 4 postpartum by Bray-Curtis dissimilarity. **e** Shannon index of stomach contents microbiota collected from pups’ stomachs 14 days after delivery. **f** Principal coordinate analysis of stomach contents microbiota by Bray-Curtis dissimilarity collected 14 days after delivery. **g** Bayesian estimates of the proportion of microbes in pup stool that came from the mother. Error bars represent standard error of the mean (SEM). Data representative of two independent experiments. *n* = 4–6 breastmilk/2 genital tract samples per group per experiment. *p < 0.05

Since vancomycin has poor oral bioavailability and would therefore not be expected to directly impact offspring microbiota, we investigated the potential indirect mechanisms through which maternal treatment could alter offspring microbiota. We first confirmed that vancomycin was indeed not absorbed by the dams nor indirectly being taken up by the pups, by measuring vancomycin levels in serum 2 days postpartum for Mg dams or 14 days in Mn/Mgn dams and Pg/Pn/Pgn pups. Vancomycin was below the detectable level of 3 μg/mL in all maternal and offspring plasma (Additional file 1: Table S1), although we cannot rule out the possibility that trace vancomycin levels below the detectable level were present. As delivery may be a determinant of offspring microbiota, and that vaginal microbiota influence infant microbiota during vaginal delivery [22, 23], we tested whether maternal gut microbiota may be influencing vaginal microbiota. Dams were treated with vancomycin for 5 days prior to delivery or left untreated (Fig. 2a). Four days postpartum, dams were sacrificed and genital tract tissue harvested. Although the Shannon index was lower in Mg, this difference was not significant (*p* = 0.066, Fig. 2b). There was also weak clustering of β-diversity in a PCoA analysis (Adonis *R*^2^ = 0.27, *p* = 0.085, Additional file 1: Figure S2A); however, the sample size was small and due to the fact that vaginal microbiota were only investigated at 4 days postpartum, we cannot rule out that differences in vaginal microbiota at delivery did not exist.

Considering the importance of breastfeeding in infant gut colonization, we next analyzed breastmilk microbiota. Given the technical challenges of collecting sufficient breastmilk for microbiota profiling directly from nursing dams, we instead sampled the pup stomach contents shortly after feeding as a proxy for the murine breastmilk. Vancomycin alteration of maternal gut microbiota resulted in significant changes in microbiota of stomach contents. In stomach contents collected at day 4 postpartum (as in Fig. 2a), significant vancomycin-dependent effects on both α- (*p* = 0.015) and β-diversity (Adonis *R*^2^ = 0.489, *p* = 0.016) were found (Fig. 2c, d). To assess the long-term effect of gestational maternal antibiotics as well as antibiotics during breastfeeding on breastmilk microbiota, stomach contents were also sampled from pups’ stomachs at day 14 postpartum (as in Fig. 1a). Here, the largest effect on both α- and β-diversity was seen in stomach contents of pups whose mothers were treated with antibiotics during nursing (Pn and Pgn; Fig. 2e). However, Pg stomach contents also showed distinct clustering from Pc (Fig. 2f).

To investigate the contribution of maternal gut and breastmilk microbiota on the establishment of pup stool microbiota, we used SourceTracker, a tool which directly estimates source proportions and uses Bayesian modeling of uncertainty about known and unknown source environments [24]. In all pups, maternal gut and stomach content microbiota had an influence on gut microbiota (Fig. 2g and Additional file 1: Figure S2B). Many of the taxa present in the Pc and Pg microbiota were from unknown sources, suggesting other sources such as skin, vaginal, or environmental microbiota have a large influence on pup microbiota. In summary, the maternal gut microbiota likely influences stomach content (as a proxy for breastmilk) and vaginal microbial composition, and together these influence bacterial colonization of the infant gut.

### Maternal gut microbiota influences inherent adaptive immunity in offspring

**Fig. 3:**
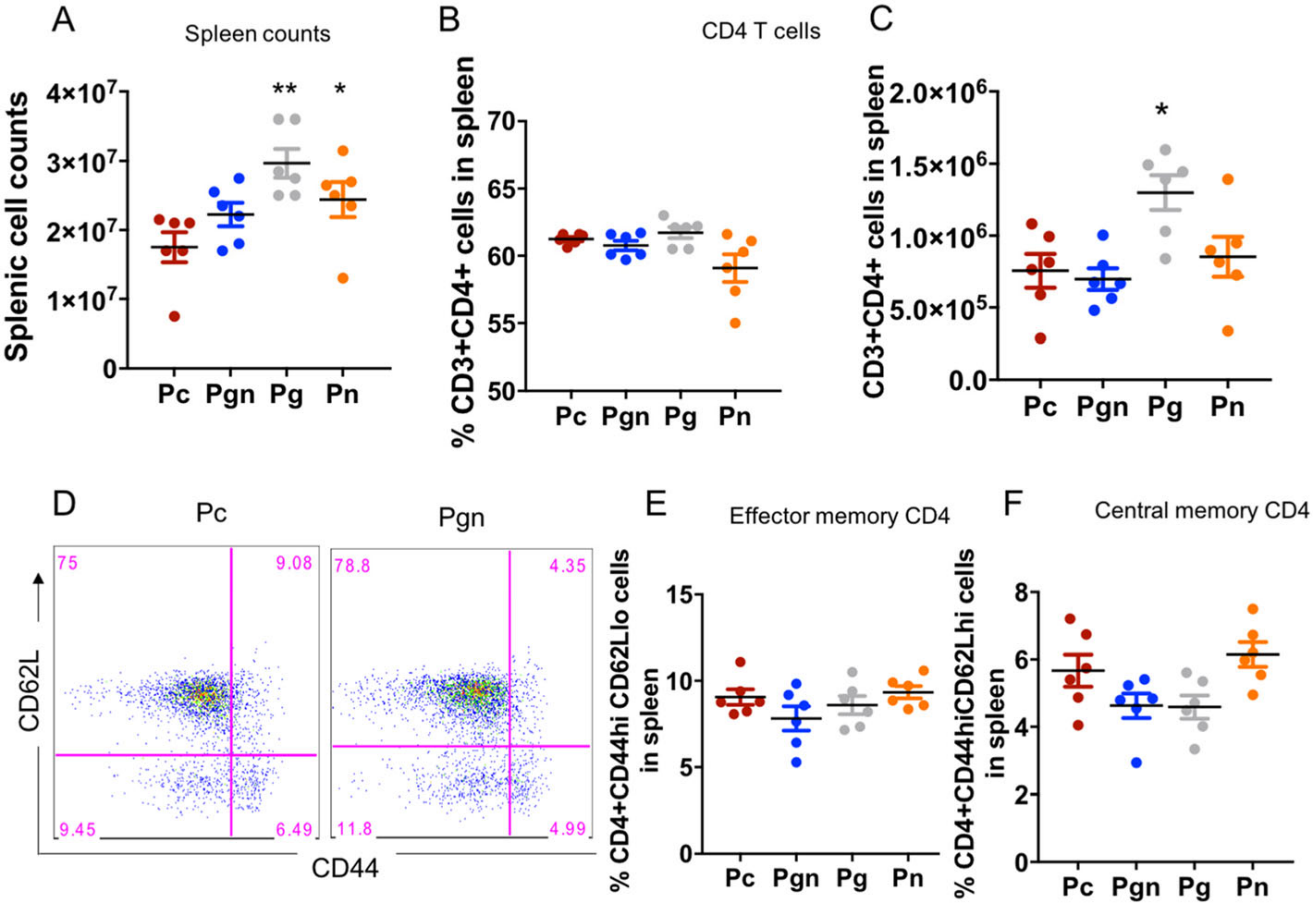
Maternal oral vancomycin impacts spleen cellularity at 14 days postpartum. Pups born to vancomycin breeders were sacrificed 14 days postpartum and their spleen lymphocyte populations characterized by FACS. **a** Total spleen cell counts of Pc (brown), Pgn (blue), Pg (gray), and Pn (yellow). **b** Proportions and **c** numbers of CD3+ cells that are CD4+. **d** Representative flow plot of effector and central memory CD4+ T cell subsets in spleen of Pc versus Pgn, gated from CD4+ population. Frequency of CD3+CD4+ that are **e** CD44hiCD62Lhi (central memory CD4 T cells) and **f** CD44hiCD62Llo (effector memory CD4 T cells). Graphs shown as mean ± SEM. Data are representative of two independent experiments. *n* = 6–10 pups per group. *p < 0.05, **p < 0.01, ***p < 0.001. Kruskal-Wallis test followed by Mann-Whitney *U* test

We next assessed whether the alterations in infant gut microbiota had consequences on lymphocyte profiles in pups. Pups born to dams treated with vancomycin had significantly higher total cell counts in the spleen compared to controls (Fig. 3a). Although no differences in proportion of CD3+ cells that were CD4+ were notable (Fig. 3b), there were significantly higher numbers of CD4+ T cells present in Pg compared to the other groups (Fig. 3c). The proportions and numbers of effector memory CD4+ T cells (CD44hiCD62Llo) and central memory CD4+ T cells (CD44hiCD62Lhi) in offspring born to mothers treated with vancomycin were similar regardless of treatment (Fig. 3e, f).

**Fig. 4:**
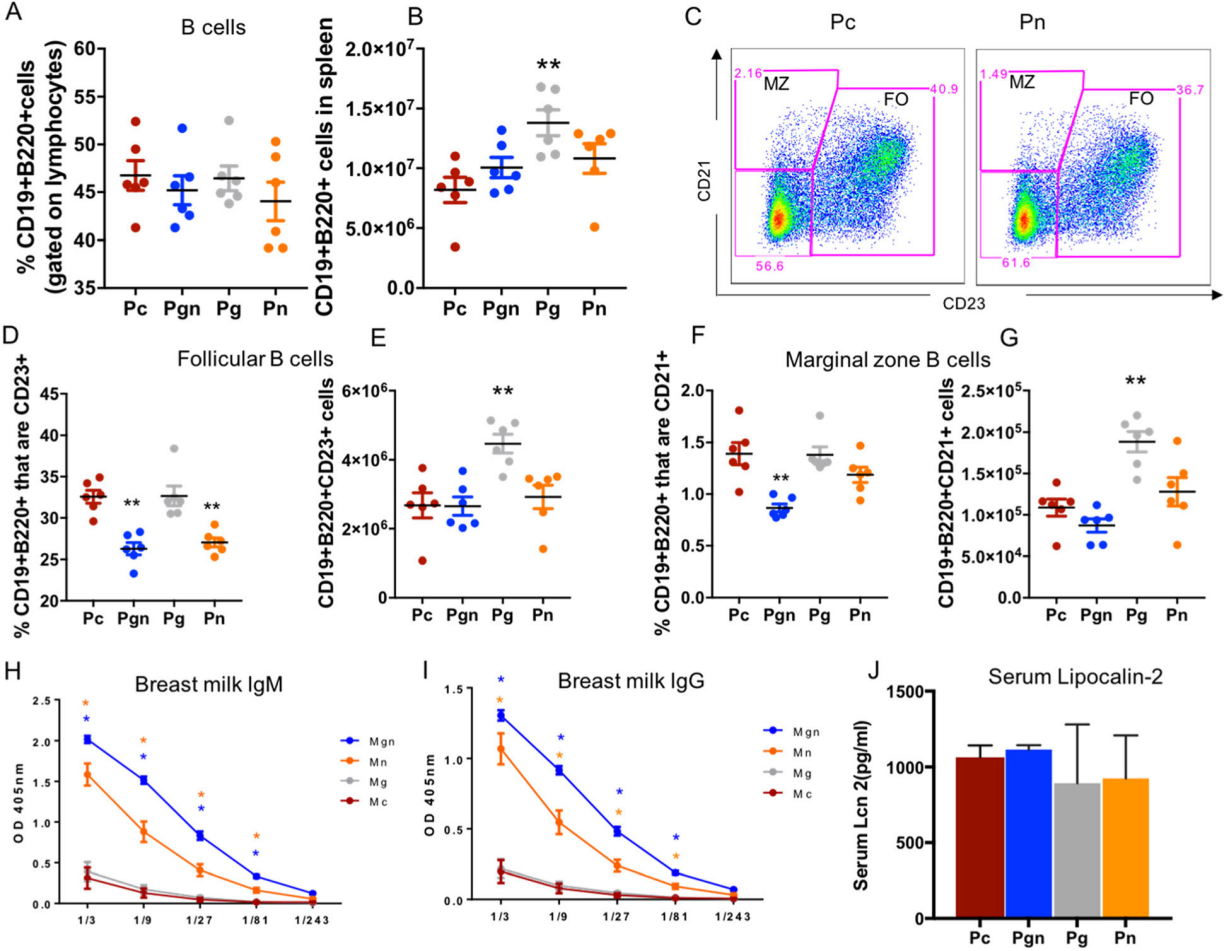
Maternal oral vancomycin impacts infant and maternal B cell compartments. Pups born to vancomycin breeders were sacrificed 14 days postpartum and their inherent spleen immunity characterized by FACS. Total IgM and IgG was measured in offspring sera and maternal breastmilk 14 days postpartum. **a**, **b** Proportion and absolute counts for total B cells. **c** Representative flow plot of Marginal and Follicular B cell subsets in spleen of Pc versus Pn. **d**, **e** Frequencies and numbers of Follicular B cells in infant spleen. **f**, **g** Frequencies and numbers of Marginal zone B cells in infant spleen. **f** Total IgM in infant serum 14 days postpartum. **h**, **i** Total IgM and IgG respectively in maternal breastmilk (collected from pup’s stomach), 14 days postpartum. **j** Serum levels of lipocalin-2 in pup serum at 14 days postpartum. Graphs shown as mean ± SEM. Data are representative of two independent experiments. *n* = 6–10 pups per groups. *p < 0.05, **p < 0.01, ***p < 0.001. Kruskal-Wallis test followed by Mann-Whitney *U* test

B cell frequencies and counts were also altered in pups of maternal vancomycin-treated groups (Fig. 4). Although there were no differences in proportions of CD19+B220+ cells in intervention groups versus Pc (Fig. 4a), Pg exhibited significantly increased numbers of CD19+B220+ cells compared with Pc (*p* = 0.020, Fig. 4b). In the follicular (FO) B cell compartment, frequency of FO cells within the B cells were significantly reduced in Pgn and Pn (*p* = 0.017 and 0.002 respectively) (Fig. 4d), and FO cell numbers were increased in Pg (*p* = 0.020). Pgn had reduced marginal zone (MZ) B cell frequency compared to controls (*p* = 0.002), whereas Pg had increased MZ B cell numbers (*p* = 0.022; Fig. 4g). Total IgG and IgM were significantly higher in stomach contents of Pgn and Pn versus Pc, implying that maternal humoral immunity may be altered in their mothers (Fig. 4h, i). To address the possibility that changes in splenic populations were due to inflammation, we measured the concentration of lipocalin-2 in serum. Lipocalin-2 has previously been described as a marker of gut inflammation [25]. We observed no difference in the concentration of serum lipocalin-2 across all pup groups (Fig. 4j) suggesting that changes in immunity were likely due to changes in microbiota and not as a result of systemic inflammation. Together, these data show that vancomycin-treatment induced changes in maternal gut microbiota associated with a significant change in breastmilk-derived antibodies as well as the profile of splenic lymphocyte populations in offspring.

## Discussion

It is established that maternal health influences offspring gut microbial colonization [26]. However, to date, few studies have investigated the effects of maternal gut microbiota during gestation on offspring immunity. In this study, we show that antibiotic alteration of maternal gut microbiota during pregnancy and/or nursing results in changes in systemic adaptive immunity in offspring.

Pup intestinal microbiota was significantly impacted by maternal vancomycin treatment, regardless of timing of intervention. No vancomycin was detectable in the serum of treated dams, suggesting that any changes in maternal microbiota at other mucosal sites, as well as in pup microbiota, were due to indirect effects of altered maternal gut microbiota and not due to direct exposure to the antibiotic. However, we cannot rule out the possibility that trace amounts of vancomycin were absorbed and directly altered the offspring microbiota. Timing resulted in unique effects on infant gut microbiota. Even when administered during gestation only, alterations in maternal gut microbial communities were evident 14 days postpartum, which were unique and clustered distinctly when compared to control- and nursing-treated dams. Furthermore, maternal gut microbial alterations during gestation affected pup stomach content (as a proxy for breastmilk) microbiota and to a lesser degree, vaginal microbiota. Microbial crosstalk between mucosal sites has been suggested to occur between surfaces such as the mouth and placenta [27], and microbial translocation from the gut to distal sites has recently been described [28].

Although breastmilk contains oligosaccharides and other prebiotics, breastmilk microbiota can influence infant gut microbiota [13]. In humans, short-chain fatty acids (SCFA), which have been implicated in immunity [29], are produced following the bacterial fermentation of human milk oligosaccharides (HMOs) that are found in breastmilk. It is possible that alterations in mouse breastmilk microbial profiles may alter the metabolite profiles in their milk, hence indirectly influencing colonization patterns in offspring. Our findings of increased total immunoglobulin G and M in stomach contents of dams who received vancomycin during nursing are interesting and consistent with a mouse model of *C. difficile* infection where vancomycin treatment was associated with lower IgA and IgG levels in sera [30]. However, these findings raise another possible mechanism through which alterations in maternal gut microbiota may influence that of the pup gut. Vancomycin-induced changes in maternal antibodies may mediate altered pup microbiota by changing the opsonized fraction of breastmilk bacteria, in addition to changing the breastmilk microbiota itself. However, since others have shown that the IgG bound fraction of breastmilk microbiota is similar to the non-bound fraction [31], this second mechanism is less likely.

Inherent immunity was altered in pups born to vancomycin-treated dams. B cells, particularly FO B cells, were significantly reduced in Pn and Pgn and increased in Pg compared to controls. These data add to the recent findings reported by Gomez and colleagues who showed no impact of maternal microbiota during pregnancy on infant T cell activation status in the bone marrow and other systemic sites [21]. However, this study utilized a germ-free maternal-neonatal model where mothers were transiently mono-colonized during gestation, an effect that was short-lived. By the time of delivery, both the maternal birth canal and the neonates were germ free. Although we did not assess long-term effects of these changes in our model, others have found long-term consequences of antibiotic-perturbed maternal microbiota on offspring susceptibility to colitis [32]. The data we present here clearly shows that an altered maternal microbiota can strikingly influence offspring adaptive immunity.

Taxonomic changes caused by maternal treatment were the likely drivers of immune outcomes in pups. A reduced relative abundance of *R. gnavus* or *B. ovatus* in Pgn and Pn could be a potential cause of reduced frequencies of FO B cells in these pups compared to controls. Therefore, it is plausible that these organisms could be exploited in future experiments to augment follicular B cell development. Alternatively, since gut microbial-derived SCFAs are important regulators of the B cell compartment and systemic immunoglobulin levels [33], these could be explored as potential immune modulators during development.

## Conclusion

In conclusion, our data provide insight into the mechanism through which maternal exposures during pregnancy are important determinants of the health of her infant. We further identify alteration of breastmilk (stomach content) microbiota as a partial intermediary between the infant and maternal gut microbiota. These findings are important since the maternal gut microbiota is potentially modifiable and therefore may be manipulated in interventions to improve infant health.

## Methods

### Experimental design

Female 6–8-week-old BALB/c mice were mated by housing two females and an adult male per cage for 7 days, after which the male was removed. Dams were treated orally with vancomycin (1 mg/mL) in drinking water 5 days prior to giving birth (gestation group), 14 days after delivery (nursing group), or 5 days prior to delivery and throughout nursing (gestation plus nursing group). Vancomycin was not administered to the control dams. We investigated the effect of oral vancomycin when administered at various phases on offspring growth, immunity, and gut microbiome. Pups were sacrificed 4 or 14 days after birth and sampled for feces (individually from colons) and breastmilk (stomach contents) for microbiome analysis and spleens for immune analysis. In dams, genital tract samples were collected 4 days after delivery for microbiome analysis.

### Sample preparations and DNA extractions

Fecal samples were collected from colons at sacrifice and stored at − 20 °C. For bacterial DNA extractions, we included an additional enzymatic lysis procedure [34] before using the Powersoil Isolation Kit (Mo Bio Laboratories). Briefly, 50 μL lysozyme (10 mg/mL, Sigma-Aldrich), 6 μL mutanolysin (25 KU/mL, Sigma-Aldrich), and 3 μL lysostaphin (4000 U/mL, Sigma-Aldrich) were added to 100 μL aliquot of cell suspension followed by incubation for 1 h at 37 °C. The lysate was then subjected to further DNA isolation and purification using the Powersoil DNA Isolation Kit (Mo Bio Laboratories) as per the manufacturer’s instructions. The final DNA concentration was determined by the Quanti-It Picogreen dsDNA HS assay kit (Invitrogen, UK).

### 16S rRNA gene sequencing

16S rRNA gene sequencing was performed using the extracted metagenomic DNA as previously described [35, 36]. Briefly, the hypervariable V6 region of the 16S rRNA gene was amplified via PCR in two steps: the first step barcoded the samples and the second added Illumina paired-end sequencing adapters [37]. The resulting PCR amplicons were purified using the Qiagen 96-well purification kit (Qiagen, CA), the amplicon concentration was determined using the Quanti-It dsDNA BR assay (Invitrogen, UK), and 50 ng from each reaction was pooled into a single tube. Pooled DNA was run on a 1.5% agarose gel and visualized, and the 330-bp band was carefully cut out of the gel and purified using a gel purification kit (Qiagen, CA). The final DNA concentration was determined and the library sequenced from both ends at The Centre for Applied Genomics at the Hospital for Sick Children in Toronto, Canada, on the Illumina HiSeq 2000 platform (100 base paired-end chemistry). Appropriate positive and negative controls were run alongside each library to confirm lack of contamination and accuracy of the analysis pipeline.

### Cell and tissue processing

Spleens were isolated aseptically and single-cell suspensions made in complete media comprising Iscove’s Modified Eagle Medium (IMDM) (Invitrogen) supplemented with 10% heat-inactivated fetal bovine serum (FBS) and 100 U/mL penicillin G and 100 μg/mL streptomycin. Single-cell suspensions were achieved by passing the organs through a 40-μm nylon cell strainer (Becton Dickson, NJ) using a 2-mL syringe plunger. Cells were then spun at 1200 rpm for 5 min and media discarded, and the red blood cells lysed by resuspending in 1 mL RBC lysis buffer (8.34 g ammonium chloride, 0.037 g EDTA and 1 g sodium hydrogen carbonate/L, pH 7.2) for 1 min. Cells were pelleted again and resuspended in complete media. Viability was determined by trypan blue exclusion. Cells were then reconstituted to a working concentration of 10^7^ cells/mL and used for culture and flow cytometry. Cells were plated in a 96-well plate and stained for expression of extracellular markers.

### Flow cytometry

Splenic lymphocytes were stained for surface markers as follows. For extracellular markers, single cells were stained at 2 × 10^6^ cells per well in a 96-well V bottomed plate. T cells were stained with anti-CD3 Alexa 700 (BD, clone 500A2), anti-CD4 PerCP (BD, clone RM4-5), anti-CD44 FITC (BD, clone IM7), anti-CD62L V450 (BD, clone MEL-14), and anti-FOXP3 APC (BD, clone MF23). B cells were stained with anti-CD19 PEcy7 (eBiosciences, clone 6D5), anti-B220 FITC (eBiosciences, clone RA3-6B2), anti-CD21 APC (BD, clone 7G6), anti-CD23 PE (BD), and anti-CD80 V450 (BD, clone 16-10A1). Fifty microliters of the antibody master mix prepared in MACS buffer (1× PBS, 2 mM EDTA, and 0.5% BSA) was added per well in all staining procedures. Cells were acquired on an LSRII (Becton Dickinson) and analyzed by FlowJo (Tree Star, Ashland).

### Flow cytometry statistical analysis

Data was summarized using routine methods [38]. Statistical analysis for mouse immunity data was performed by GraphPad Prism version 6. Comparisons were made by non-parametric analysis of variance followed by Mann-Whitney *U* test. Data were considered statistically significant if *p* < 0.05.

### Antibody ELISAs

Breastmilk pellets were collected from pups’ stomach 2 weeks postpartum. Relative levels of IgG or IgM levels were determined by antibody ELISA. Briefly, breastmilk pellets were homogenized in 200 μl PBS. Samples were then spun at 4000 rpm for 10 min and supernatants collected. Protein concentrations were determined by BCA assay and normalized to equal concentrations. Flat-bottomed plates (Nunc, Maxisorp) were coated with 50 μl of IgG or IgM capture antibody and incubated overnight at 4 °C. The next day, plates were washed and blocked with 200 μl/well of 4% BSA in PBS for 3 h at 37 °C. After washing three times, samples were added. Samples were diluted serially across six wells starting with a dilution of 1.3. Plates were then incubated overnight before being washed and the detector antibody (50 μl/well) added (Southern Biotech) for 3 h at 37 °C. Plates were then washed and the signal was detected using substrate p-nitrophenylphosphate powder at 1 mg/ml (Sigma-Aldrich). Plates were incubated at 37 °C until the desired coloration was obtained and read at a wavelength of 405 nm using the Softmax Pro Program. Graphs were plotted as dilutions versus optical densities.

### Lipocalin-2 ELISA

Serum concentrations of lipocalin-2 were determined using the Mouse Lipocalin-2/NGAL Quantikine ELISA kit (R & D Systems, Minneapolis, MN). Samples were diluted 1:100, and assay was performed according to the manufacturer’s instructions. Samples were assayed in duplicate.

### Microbiome analysis

Sequence data was pre-processed in QIIME and UPARSE [39, 40]. Briefly, sequences lacking barcodes were removed and samples with less than 100,000 reads discarded. PCR errors were removed by SeqNoise [41]. Primers and barcodes were removed from de-noised sequences. Consequently, de-noised sequences were clustered into operational taxonomic units (OTUs) at 97% sequence similarity. Taxonomic assignment was done by RDP classifier using the Greengenes database (version 13.8). The biom file was then imported into Calypso or R for further downstream analysis.

Statistical analysis was done using Calypso version 6.4 using the default parameters [42]. Analyses were performed by Kruskal-Wallis and Wilcoxon rank test and *p* values adjusted for multiple comparisons by Benjamini-Hochberg false discovery rate (FDR). Additional analyses were performed in R, using the R packages phyloseq for beta diversity analyses [43], vegan [44] for ordination and redundancy analysis, and randomForest [45] for predictive modeling. Statistical testing was corrected for false discovery rate (FDR) by Benjamini-Hochberg method, and adjusted *p* values less than 0.05 were considered statistically significant. Differences in microbial composition between groups of interest were assessed using metagenomeSeq’s MRfulltable function [44] with a custom filter to determine significance: taxa were deemed significantly different if they exhibited a fold change (beta coefficient) of ≥ 1.25, if they had an adjusted *p* value of ≤ 0.05, and if at least one of the two groups being compared had ≥ 20% of samples with the given OTU/taxa OR Fisher’s exact test result was significant (after multiple testing correction using the Benjamini-Hochberg method). These results were confirmed using the DESeq2 package [46]. To investigate the contribution of maternal gut and breastmilk microbiota to the establishment of offspring gut microbiota, we used a Bayesian approach for bacterial source tracking as has been previously described [24]. Pup fecal samples were designated as sinks and maternal samples (gut and breastmilk) of the corresponding mother selected as sources.

## Additional file

Additional file 1: **Figure S1.** (A) Shannon α-diversity of maternal fecal microbiota. (B) of maternal fecal microbial β-diversity based on Bray Curtis distance. (C) Relative abundance at phyla level in dams. (D) PCoA of both pup and dam stool microbiota in different cages. Results were combined from two independent experiments. Related to Fig. 1. **Figure S2.** Maternal gut microbiota and breast milk microbiota influence pup gut microbiota. Genital tract samples were collected day 4 post delivery from dams (A) Principal coordinate analysis by Bray-Curtis dissimilarity of genital tract microbiota. (B) Pie charts showing representative pie charts of maternal source of bacteria in individual infant mice gut. Data representative of two independent experiments. *n* = 4 genital tract samples per group or 4–6 pups per group. *p < 0.05. Related to Fig. 2. **Table S1.** Vancomycin levels in serum of dams and pups measured by the Abbott ELISA technique. Levels across all groups were below the detection limit of 3 μg/ml. Related to Fig. 1. (DOCX 773 kb)
