# Supplementary material for: Disruption of maternal gut microbiota during gestation alters offspring microbiota and immunity
Source: Microbiome. 2018 Jul 7;6:124. doi: 10.1186/s40168-018-0511-7 (PMC6035804; doi:10.1186/s40168-018-0511-7)
Supplement: Supplementary file 1 — Figure S1. (A) Shannon α-diversity of maternal fecal microbiota. (B) of maternal fecal microbial β-diversity based on Bray Curtis distance. (C) Relative abundance at phyla level in dams. (D) PCoA of both pup and dam stool microbiota in different cages. Results were combined from two independent experiments. Related to Fig. 1. Figure S2. Maternal gut microbiota and breast milk microbiota influence pup gut microbiota. Genital tract samples were collected day 4 post delivery from dams (A) Principal coordinate analysis by Bray-Curtis dissimilarity of genital tract microbiota. (B) Pie charts showing representative pie charts of maternal source of bacteria in individual infant mice gut. Data representative of two independent experiments. n = 4 genital tract samples per group or 4–6 pups per group. *p < 0.05. Related to Fig. 2. Table S1. Vancomycin levels in serum of dams and pups measured by the Abbott ELISA technique. Levels across all groups were below the detection limit of 3 μg/ml. Related to Fig. 1. (DOCX 773 kb) [file 40168_2018_511_MOESM1_ESM.docx]

**Additional file Figures and Tables**

**Figure S1**: **(A)** Shannon α-diversity of maternal fecal microbiota. **(B)** of maternal fecal microbial β-diversity based on Bray Curtis distance. **(C)** Relative abundance at phyla level in dams. (D) PCoA of both pup and dam stool microbiota in different cages. Results were combined from two independent experiments. Related to **Figure 1.**

**Figure S2:** **Maternal gut microbiota and breast milk microbiota influence pup gut microbiota.** Genital tract samples were collected day 4 delivery from dams **(A)** Principal coordinate analysis by Bray-Curtis dissimilarity of genital tract microbiota. **(B)** Pie charts showing representative pie charts of maternal source of bacteria in individual infant mice gut. Data representative of two independent experiments. n=4 genital tract samples per group or 4-6 pups per group. *p <0.05. Related to **Figure 2.**

**Table S1**: Vancomycin levels in serum of dams and pups measured by the Abbott ELISA technique. Levels across all groups were below detection limit of 3µg/ml. **Related to Figure 1**.

**Figure S1**


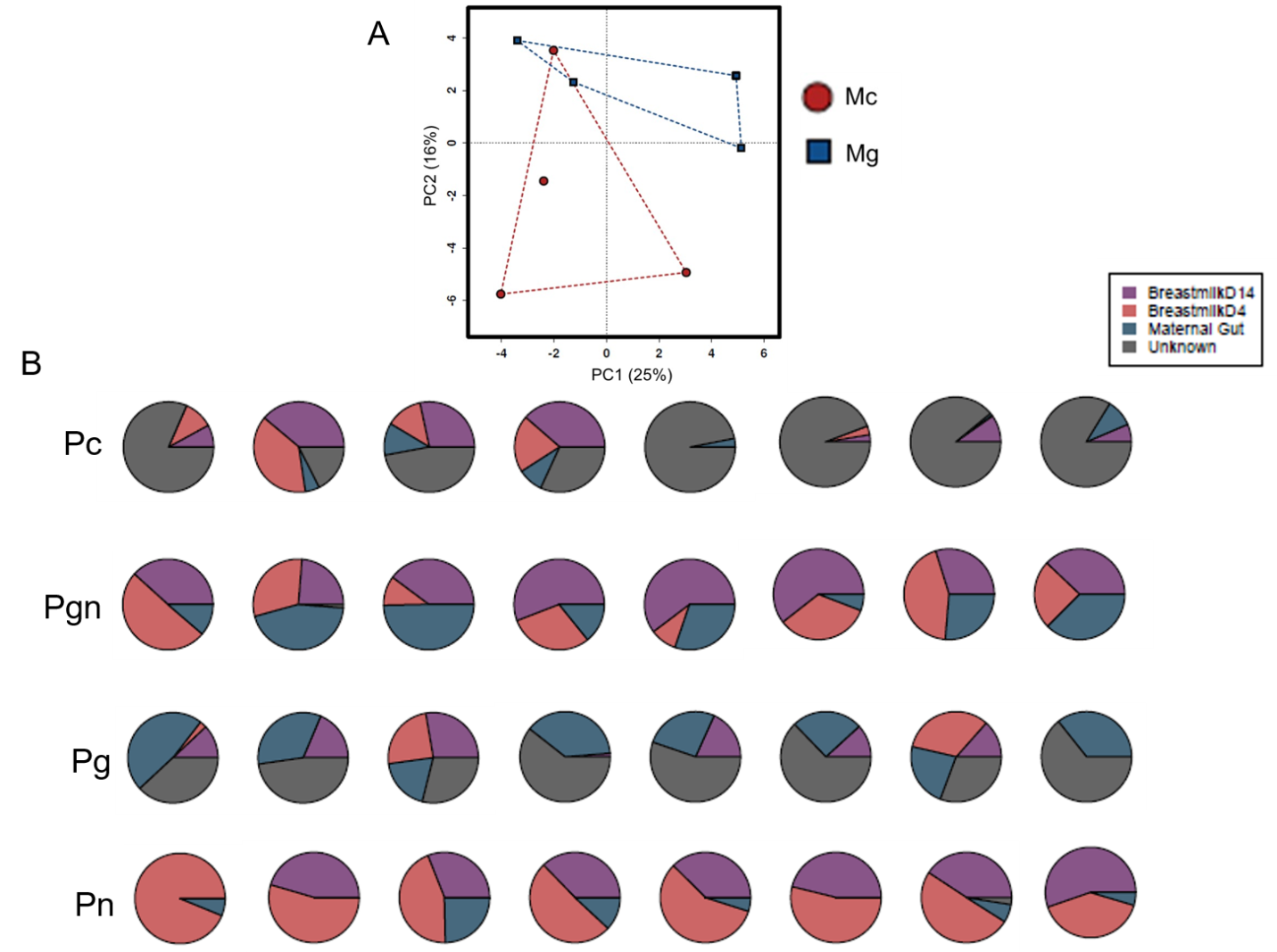


**Figure S2**

**Table S1**

| Sample ID | Result |
| --- | --- |
| Pg1 | <3µg/ml |
| Pg2 | <3µg/ml |
| Pg3 | <3µg/ml |
| Pg4 | <3µg/ml |
| Pn1 | <3µg/ml |
| Pn2 | <3µg/ml |
| Pn3 | <3µg/ml |
| Pn4 | <3µg/ml |
| Pgn1 | <3µg/ml |
| Pgn2 | <3µg/ml |
| Pgn3 | <3µg/ml |
| Pgn4 | <3µg/ml |
| Mg | <3µg/ml |
| Mg | <3µg/ml |
| Mn | <3µg/ml |
| Mn | <3µg/ml |
| Mgn | <3µg/ml |
| Mgn | <3µg/ml |
| Positive control 1 | 5.5µg/ml |
| Positive control 2 | 22µg/ml |
| Positive control 3 | 49µg/ml |
